# Chlamydia trachomatis genomes from rectal samples: description of a new clade comprising ompA-genotype L4 from Argentina

**DOI:** 10.1099/mgen.0.001350

**Published:** 2025-02-13

**Authors:** Karina Andrea Büttner, Fanny Wegner, Vera Bregy, Andrea Carolina Entrocassi, María Lucía Gallo Vaulet, Deysi López Aquino, Luciana La Rosa, Laura Svidler López, Mirja H. Puolakkainen, Eija Hiltunen-Back, Frank Imkamp, Adrian Egli, Helena M.B. Seth-Smith, Marcelo Rodríguez Fermepin

**Affiliations:** 1 Universidad de Buenos Aires, Facultad de Farmacia y Bioquímica, Departamento de Bioquímica Clínica, Cátedra de Microbiología Clínica, Buenos Aires, Argentina; 2 Universidad de Buenos Aires, Instituto de Fisiopatología y Bioquímica Clínica (INFIBIOC), Buenos Aires, Argentina; 3 Member of the ESCMID study Group on Mycoplasma and Chlamydia (ESGMAC); 4 Institute of Medical Microbiology, University of Zürich, Zurich, Switzerland; 5 División Cirugía, Hospital Fernández, Buenos Aires, Argentina; 6 Centro Privado de Cirugía y Coloproctología, Buenos Aires, Argentina; 7 University of Helsinki, Department of Virology and Helsinki University Hospital, Department of Virology and Immunology, Helsinki, Finland; 8 Department of Dermatology and Allergology, University of Helsinki and HUS Helsinki University Hospital, Helsinki, Finland

**Keywords:** L2b, Phylogeny, STI, whole genome sequencing

## Abstract

Whole-genome analysis has provided insights into the evolution of *Chlamydia trachomatis* and, recently, into circulating strains that cause lymphogranuloma venereum (LGV). A large LGV outbreak of a new *ompA*-genotype, L2b, was first reported in Europe in the early 2000s, primarily affecting men who have sex with men (MSM), and then expanded globally. More recent work shows that this outbreak is diversifying into variants of described *ompA*-genotypes, with the same L2b genomic backbone. This study extends the investigation of LGV cases to Argentina and Finland. In 2017, an LGV outbreak was described in Argentina characterized by distinct genomic features shown by both *ompA*-genotyping and Multi-Locus Sequence Typing (MLST) analysis. We have obtained whole-genome sequences from cultured isolates and clinical samples via SureSelect (Agilent) target enrichment. Based on *ompA* and phylogenetic analyses, we describe further diversity within the *ompA*-genotype L2b clade, illustrating the transmission dynamics in Argentina and Finland. A key finding is that of a novel clade of Argentinian samples, characterized by a proposed new *ompA*-genotype L4. Additionally, we present the genome sequence of a non-LGV strain associated with anorectal proctitis. These findings contribute to the investigation of LGV evolution, particularly with the presence of the novel L4 lineage, and provide insights into genomic diversity and transmission dynamics of *C. trachomatis*.

## Graphical Abstract

We describe a new lineage of *Chlamydia trachomatis* lymphogranuloma venereum identified in Argentina, carrying *ompA*-genotype L4. We also present further diversification of the globally circulating L2b lineage.

**Impact Statement**

The phylogeny of *Chlamydia trachomatis* has been described in several publications and has been clear for a decade. The evolution of this sexually transmitted infection in the intervening years also informs a great deal about the transmission opportunities and selective pressures that the intracellular bacterium is under. Through genome sequencing, also directly from clinical samples, we provide the first lymphogranuloma venereum (LGV) genomes from Argentina and Finland and describe further evidence of global circulation of the *ompA*-genotype L2b lineage. We describe a fully novel lineage of LGV *C. trachomatis*, from Argentina, proposed as *ompA*-genotype L4. We also find evidence of a strain causing proctitis from the urogenital lineage. Together, these provide significant new findings in the investigation of *C. trachomatis*.

## Data Summary

All Illumina sequence data, with human read data removed using Hostile [1] and KrakenTools (https://github.com/jenniferlu717/KrakenTools), are deposited with the European Nucleotide Archive under project number PRJEB72167 with the accession numbers given in Table S1. The Supplementary Material can be found at https://doi.org/10.6084/m9.figshare.28131641.v1 [2].

## Introduction

The sexually transmitted infection (STI)-causing bacterium *Chlamydia trachomatis* has an invasive biovar, lymphogranuloma venereum (LGV), which causes LGV, as well as the urogenital (UG) and ocular biovars. Recently, interest has arisen around the current evolution of LGV strains of *C. trachomatis* [3,5]. Whole-genome sequencing is the best method to further investigate globally circulating strains of *C. trachomatis* LGV.

The most recent genomic findings on LGV *C. trachomatis* showed ongoing evolution of the *ompA*-L2b lineage, with the few genomic SNPs found over-represented in the *ompA* gene, generating novel variations in the encoded Major Outer Membrane Protein (MOMP) protein [3].

Comprehensive nationwide prevalence data for *C. trachomatis* are lacking in Argentina, as is the identification of all LGV cases; however, the limited data suggest higher rates of LGV in Buenos Aires than in Europe [6,8]. Since 2017, proctitis patients in Buenos Aires, mostly Human Immunodeficiency Virus (HIV)-infected MSM, have been screened for *C. trachomatis* [9], showing a range of *ompA*-genotypes [10] and MLST types [11].

This work further investigates LGV genomes from Argentina and Finland in the context of global data to broaden the spectrum of LGV genomes analysed to date. We show further developments in the *ompA*-genotype L2b lineage, confirm the genomic separation of a new South American lineage, termed *ompA*-genotype L4, and describe the context of a rectally derived UG lineage strain.

## Methods

### Sample collection, culture and DNA extraction

Rectal swab samples were obtained from symptomatic adult patients presenting proctitis-compatible symptoms and STI risk factors between September 2017 and August 2023, attending a public hospital and a private clinic in Buenos Aires, Argentina (Hospital Juan A. Fernández and Centro Privado de Cirugía y Coloproctología). Additionally, rectal swab samples from symptomatic patients and those with partner notifications attending the outpatient STI clinic at the Helsinki University Central Hospital in Finland between 2011 and 2013 were collected from MSM with symptoms suggestive of LGV or due to partner notification. These samples were either cultured or had DNA extracted as described in [12].

### Sequencing

Genomic sequencing data were obtained either from cultured samples or via SureSelect target enrichment (Agilent) (Table S1). Methods were performed as described in [12]. Briefly, target enrichment was performed on clinical samples with SureSelect XT HS2 DNA with post-capture pooling for Illumina Platform NGS (G9983-90500 Rev A0, Agilent SureSelect), using baits designed to cover all diversity of *C. trachomatis* using Design ID S3465202 and Design ID S3465164. Samples were sequenced PE150 on Illumina MiSeq or NextSeq1000 platforms.

### Sequence analysis

Reads were trimmed with trimmomatic v0.39 [13]. Mapping to reference AM884177, base calling and generation of multiple sequence alignments used snippy v4.6.0 (https://github.com/tseemann/snippy) with default parameters, except a minimum coverage of 5 to enable calling from regions of lower read depth, and with the rRNA operons masked. Genomes were considered acceptably complete when the percentage of genome covered was >95%, the mean read depth was >10 and <20% Ns were called at <0–5× read depth. The presence of more than one strain of *C. trachomatis* in all samples was analysed using the R package vcfR [14] by looking at all minority variants with a minimum frequency of 10% and a minimum depth of 5. All were found to have fewer than 200 minority variants and are thus considered pure strains.

Phylogenies were created using all LGV genome data available [3,15, 16] in IQtree v2.2.0.3 [17] with 1000 ultrafast bootstraps. Gubbins v3.3.0 [18] was used to remove recombinations, with five iterations, 1000 bootstraps, iqtree-fast as first-tree-builder, iqtree as tree-builder and custom model K3Pu+F+I+I+R3. FastBAPS was used to define clades (https://github.com/gtonkinhill/fastbaps). UG phylogenies were built by comparing with previous UG genomes and mapping against the genome of E/Bour (HE601870) [15]. Phylogenies were visualized with associated metadata in Phandango [19]. The whole-genome alignments are given in Files S1 and S2 available in the online Supplementary Material.

Complete coding sequences (CDSs) of *ompA* were extracted from the mapped alignments and compared with capillary sequence data [11] and blastn results from the National Center for Biotechnology Information (NCBI) database (https://blast.ncbi.nlm.nih.gov/Blast.cgi?) in AliView v1.27 [20] (Table S1).

### Statistical analysis

Statistical analysis of patient data was performed using InfoStat software version 2020, Universidad Nacional de Córdoba, Córdoba, Argentina. The two-tailed Fisher exact test was used for categorical variable analysis. Statistical significance was defined as *P*<0.05.

## Results and discussion

### Phylogeny of *C. trachomatis* LGV genomes

**Fig. 1.**
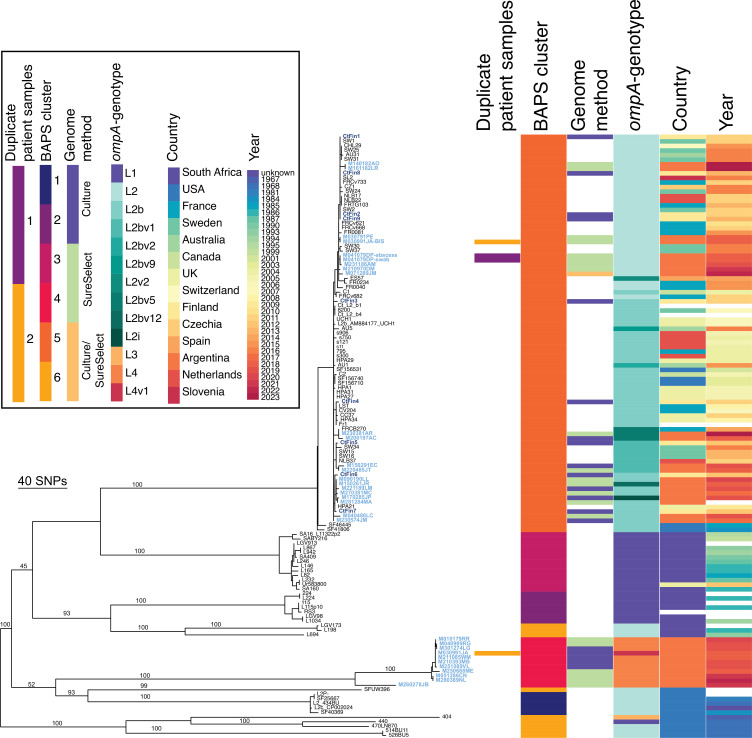
Recombination-adjusted phylogeny of LGV strains from Argentina, Finland and previous publications. All published LGV genomes with available data were included in the analysis. The right of the phylogeny is relevant metadata: duplicate patient samples, Bayesian Analysis of Population Structure (BAPS) cluster (clade, with the polyphyletic BAPS cluster 6 representing the genetic diversity of the population), genome method (data for this paper), *ompA*-genotype, country and year as described in the key to the top left. Missing data are displayed as white. Names of genomes from this publication are coloured in light blue for Argentina and dark blue for Finland in the phylogeny. Bootstrap values from 1000 ultrafast bootstraps are shown as percentages on major branches.

*C. trachomatis* LGV genomes (*n*=32 from Argentina and *n*=9 from Finland) were obtained following either culture (*n*=10 from Argentina and *n*=9 from Finland) or SureSelect (*n*=22 from Argentina, one of which had been cultured but required target enrichment to produce a full genome) and Illumina sequencing. These are the first genomes from Argentinian and Finnish LGV strains. The genomes were compared phylogenetically with previously published LGV genomes (Fig. 1). A phylogeny with duplicate samples (*n*=8), showing the reproducibility of the SureSelect method, is given in Fig. S1, and the recombinations identified within the LGV genomes are shown in Fig. S2.

### Further global circulation of the *ompA*-genotype L2b lineage

Many (*n*=21/32) of the Argentinian samples and all the Finnish samples (*n*=9) fall within the *ompA*-genotype L2b clade (BAPS cluster 5 in Fig. 1). The samples within this L2b clade show diverse *ompA*-genotypes even over short time spans, suggesting selective pressure on the encoded MOMP protein. Several sub-clades are apparent, with six separate locations of the Finnish samples in the phylogeny speaking for European circulation of many strains. Three pairs of samples represent the possible local circulation of strains in Helsinki: these are unlikely to be from direct partners or through partner notifications due to disparate sampling dates.

The closely related groups of two to six genomes from Argentinian samples suggest locally circulating strains among the MSM community in Buenos Aires. One patient had two genomically identical samples from two different locations (an abscess and an anorectal swab), both being *ompA*-genotype L2 within the L2b clade. It is worth noting that the other samples in sub-clades were independent and, based on the patients’ histories, were likely not connected. These groups are often closely related to samples from Europe, indicating multiple importations of strains from diverse international sources. As these are the most recent samples in the phylogeny, ongoing travel is not yet apparent. Of note, two Argentinian samples from 2017 and 2023, respectively, carry identical genomes to several European samples, including L2b/UCH1 and L2b/UCH2 from 2006, reiterating that *C. trachomatis* has a less clock-like mutation rate than other bacterial species [15]. The L2b clade is clearly highly international, reflecting a global circulating lineage of *C. trachomatis* LGV.

Complete genomes were obtained from two samples carrying new *ompA* variants (L2i and L2bv12) recently described in Argentina [11]: the *ompA* variant L2i, carrying a mutation that leads to an S162I substitution at the site that differentiates *ompA*-genotypes L2 and L2b, and L2bv12, carrying a mutation that leads to the S164D substitution, both in the variable domain (VD)2.

### Description of novel lineage from Argentina

We identified a new lineage comprising 11 Argentinian samples. This separate clade (BAPS cluster 4 in Fig. 1) is defined by a branch of >200 SNPs and is confirmed as a separate genetic cluster. Of interest, one patient had two samples taken over time: *ompA*-genotype L4 from April 2018 and *ompA*-genotype L2 within the L2b clade from August 2018, suggesting reinfection with a different strain after treatment (Fig. 1).

The *ompA* gene of the L4 clade is most similar to that of *ompA*-genotype L1 (accession HE601950.1), but with ten nucleotide substitutions; two versions of *ompA*-genotype L4 (L4 and L4v1) vary by a single SNP [11] (Table S1). The amino acid substitutions compared with *ompA*-genotype L1 are all located in VDs: A90T in VD1, N162S in VD2 and T311A in VD4. Taking into account this level of diversity from all previously described LGV genomes and the number of samples defining the clade (*n*=11), we propose the new *ompA*-genotype L4.

This novel lineage shows a tight sub-clade of strains presumably representing an ongoing outbreak, with a more distantly related member also represented towards the root of the clade, showing the diversity present in the Buenos Aires area. There is likely to be further unsampled diversity within this clade, perhaps with more diverse members of this clade in other areas of South America. The lineage also appears to be present in France [21]: genomics could here give insights into the origins of this lineage.

### Clinical relevance of *ompA*-genotype L4 lineage

Comparative analysis of the clinical data between the patients in clades L2b and L4 shows differences in the frequencies of some factors, although without statistical significance (Table 1). The presence of an anorectal inflammatory tumour was higher in patients infected with LGV from the *ompA*-genotype L4 clade (50% compared with 25% in the *ompA*-genotype L2b clade). The presence of anal ulcers was more frequent in patients infected with LGV from the *ompA*-genotype L2b clade (55% compared with 22% in the *ompA*-genotype L4 clade), and the presence of other concomitant STIs in addition to HIV was higher in LGV from the *ompA*-genotype L2b clade (50% compared with 30% in the *ompA*-genotype L4 clade). However, the number of cases analysed is too low to draw meaningful conclusions.

**Table 1.** Clinical data of patients from Buenos Aires. Comparison of symptoms between LGV from clades *ompA*-genotype L2b and *ompA*-genotype L4

Sample	LGV clade	Rectal syndrome*	Anal ulceration†	Severity of proctitis	Inguinallymphadenopathy	Tumour‡	HIV	Other STI
M010179RR	L4	+	−	Moderate	−	−	+	−
M030991JA	L4	+	−	Moderate	+	Anorectal	+	GC/HPV/TP
M040989RG	L4	+	−	Severe	+	Anorectal	+	−
M051286CN	L4	+	+	Moderate	−	−	−	HSV
M210393MB	L4	+	−	Moderate	+	Anorectal	+	−
M211085WM	L4	+	−	Not verified§ **^/^**	−	−	−	−
M250688ME	L4	+	+	Mild	+	Anorectal	+	−
M251089VL	L4	+	−	Moderate	+	−	+	−
M260278JB	L4	+	−	Severe	+	Anorectal	+	TP
M280389NL	L4	+	nd	Moderate	+	−	+	−
M030791PE	L2b	+	+	Severe	+	−	+	TP/HPV
M030991JA-BIS	L2b	+	−	Moderate	+	Anorectal	+	NG/TP/HPV
M040486LC	L2b	+	−	Moderate	−	−	+	−
M041079DP	L2b	−	+	Not verified§	+	nd	+	−
M071289JM	L2b	nd	+	Mild	−	−	+	−
M090190LL	L2b	+	+	Moderate	+	−	+	TP
M140182AO	L2b	+	−	Moderate	−	−	−	−
M150261JR	L2b	−	−	Moderate	−	−	+	NG/HPV
M150291EC	L2b	+	+	Severe	−	−	+	−
M161182LR	L2b	+	−	Moderate	+	−	+	−
M170285JP	L2b	+	−	Moderate	+	−	+	HPV/HSV
M200197AC	L2b	+	−	Mild	+	Anorectal	+	NG/TP/HSV
M210970DM	L2b	+	−	Severe	+	−	+	−
M220485JT	L2b	+	+	Moderate	−	−	+	−
M221189LM	L2b	+	−	Moderate	+	−	+	NG
M230381AR	L2b	+	+	Moderate	+	−	−	−
M230574JM	L2b	+	+	Severe	−	−	+	−
M231186AM	L2b	+	+	Severe	+	Anorectal	+	NG
M270381MC	L2b	+	+	Not verified§	+	Endoanal	+	HPV
M281284MA	L2b	−	+	Severe	−	Perianal	+	TP
*P*-value L4/L2b		0.15	0.11	0.82	0.55	0.12	0.65	0.26

* Rectal syndrome is characterized by rectal discharge, pushing and tenesmus.

† Ulcers located in the anal region.

‡ A mass located around the anal area

§ Not verified, as rectoscopy was not performed.

nd, Not done.

HPV, human papillomavirus; HSV, herpes simplex virus; NG, Neisseria gonorrhoeae; TP, Treponema pallidum.

### Urogenital T1 lineage sample causing proctitis

**Fig. 2.**
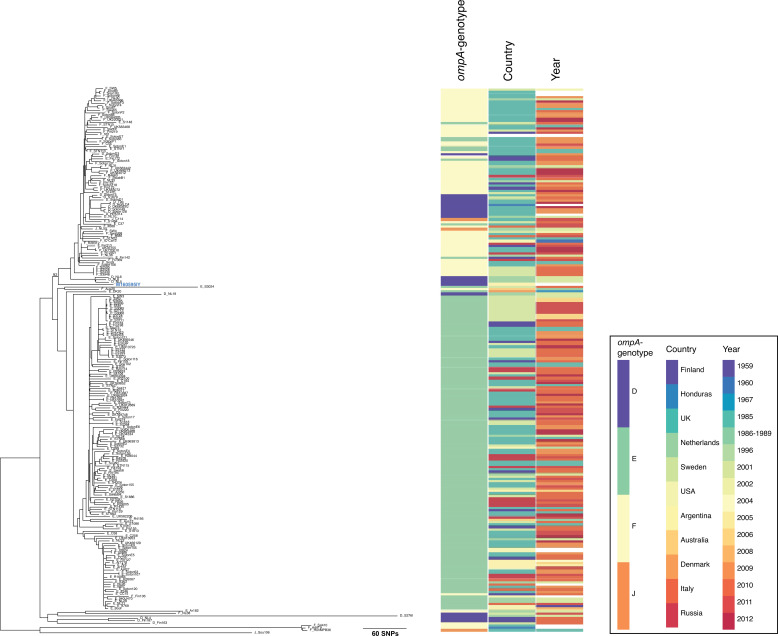
Recombination-adjusted phylogeny of UG strains belonging to lineage T1 [15] showing the location of sample M160595IY from Argentina in the context of those from previous publications. The tree is rooted according to [15]. The right of the phylogeny is relevant metadata: *ompA*-genotype, country and year as described in the key to the far right. The name of the genome from this publication is pale blue in the phylogeny. The bootstrap of 92% from 1000 ultrafast bootstraps is shown on the branch of interest.

One sample from Argentina falls within the UG T1 lineage, as described by Hadfield *et al.* [15] (Fig. 2). Sample M160595IY is located at the base of a clade of diverse genomes from diverse countries. Sample M160595IY was identified as *ompA*-genotype D by RFLP, and from Whole Genome Sequencing (WGS), it is 1 bp (synonymous SNP at amino acid 212) from other described D strains (Table S1). This branch is also defined by several recombination events, covering the same loci that are often affected by recombination in the T1 lineage strains (data not shown).

Sample M160595IY was taken from a young man presenting with symptoms of mucorrhoea, proctalgia and tenesmus, which had been developing for a month prior to the medical consultation. Diagnosis revealed mild proctitis. The patient’s sexual practices include both receptive and insertive anal sex, as well as oro-anal sex, with a reported 20 sexual partners in the previous 12 months. The patient did not present any other bacterial STI coinfections at the time of symptom onset and responded to the antimicrobial treatment. That the genome derives from an anorectal swab from a patient with proctitis, with no evidence of a mixed infection with an LGV strain in the sample, suggests that this UG strain was the cause of mild proctitis.

Genome sequences from *ompA*-genotypes E, G and Ja isolated from rectal swabs have previously been published [22,23], and non-LGV genotypes causing proctitis in patients have been identified through *ompA*-genotyping [7,24]. Additionally, recombinant LGV/UG strains have been identified in the past [4,25, 26]; however, in this case, we see a genome with *ompA*-genotype D in a UG genomic backbone isolated from an anorectal swab from a male patient with proctitis. This is the first genome sequenced from a symptomatic proctitis case caused by a non-recombinant *ompA*-genotype D isolate [7,22,24]. A deeper investigation into such samples may lead to the identification of genomic regions involved in rectal tropism.

### Plasmid analysis

Plasmids from the new strains were also analysed in the context of previously published data. Among the LGV plasmids, there is very little diversity, and the plasmid is highly conserved. All the plasmids from the novel *ompA*-genotype L4 clade are located very closely in the phylogeny, with the plasmids from L1/440, L2/514BU11 and L2/526BU5, all from the USA and isolated in 1968 (Fig. S3A). This could indicate plasmid exchange between *ompA*-genotypes within the Americas, although further data would be required to confirm this. While the plasmids of many strains within the *ompA*-genotype L2b clade are almost identical to that in L2b/UCH2, one plasmid (from sample M230574JM) stands out for having a single, synonymous SNP in plasmid coding sequence *CDS1*.

The plasmid from the UG strain M160595IY is located in the phylogeny close to many plasmids from *ompA*-genotypes D, E, F and J (Fig. S3B). It carries three additional SNPs, representing synonymous mutations in *CDS1* and *CDS8*, and R324I in *CDS3*. This would be consistent with the genomic phylogenetic position.

### Conclusions

This study demonstrates the widespread global distribution of the genomic clade of *ompA*-genotype L2b, its success as the primary causative agent of LGV and the recently evolved diversity within the clade. It confirms that in locations distant from the most studied European part of the outbreak such as Argentina, strains identified as L2 by *ompA*-genotyping also present the genetic backbone of *ompA*-genotype L2b. Our data suggest that in Buenos Aires, there have been multiple introduction events of this *ompA*-genotype L2b clade.

We also present a complete genome of a non-LGV *ompA*-genotype D sample causing proctitis, confirming their UG genomic backbone. This highlights the fact that some non-LGV strains can cause proctitis that in some cases is symptomatically similar to that produced by LGV strains.

A further key finding is the detection of a new clade causing LGV in Buenos Aires, which is vastly different from the previously described lineages, leading us to propose its designation as *ompA*-genotype L4. Future studies will further investigate the diversity within this clade and the association of the new *ompA*-genotype L4 clade with possible specific clinical manifestations.

## Supplementary material

10.1099/mgen.0.001350 Uncited Supplementary Material 1.

## Abbreviations

BAPS: bayesian analysis of population structure
HIV: human immunodeficiency virus
LGV: lymphogranuloma venereum
MLST: multi-locus sequence typing
MOMP: major outer membrane protein
MSM: men who have sex with men
NCBI: National Center for Biotechnology Information
STI: sexually transmitted infection
UG: urogenital
