# Supplementary material for: Chlamydia trachomatis genomes from rectal samples: description of a new clade comprising ompA-genotype L4 from Argentina
Source: Microb Genom. 2025 Feb 13;11(2):001350. doi: 10.1099/mgen.0.001350 (PMC12282313; doi:10.1099/mgen.0.001350)
Supplement: Uncited Supplementary Material 1. [file mgen-11-01350-s001.pdf]

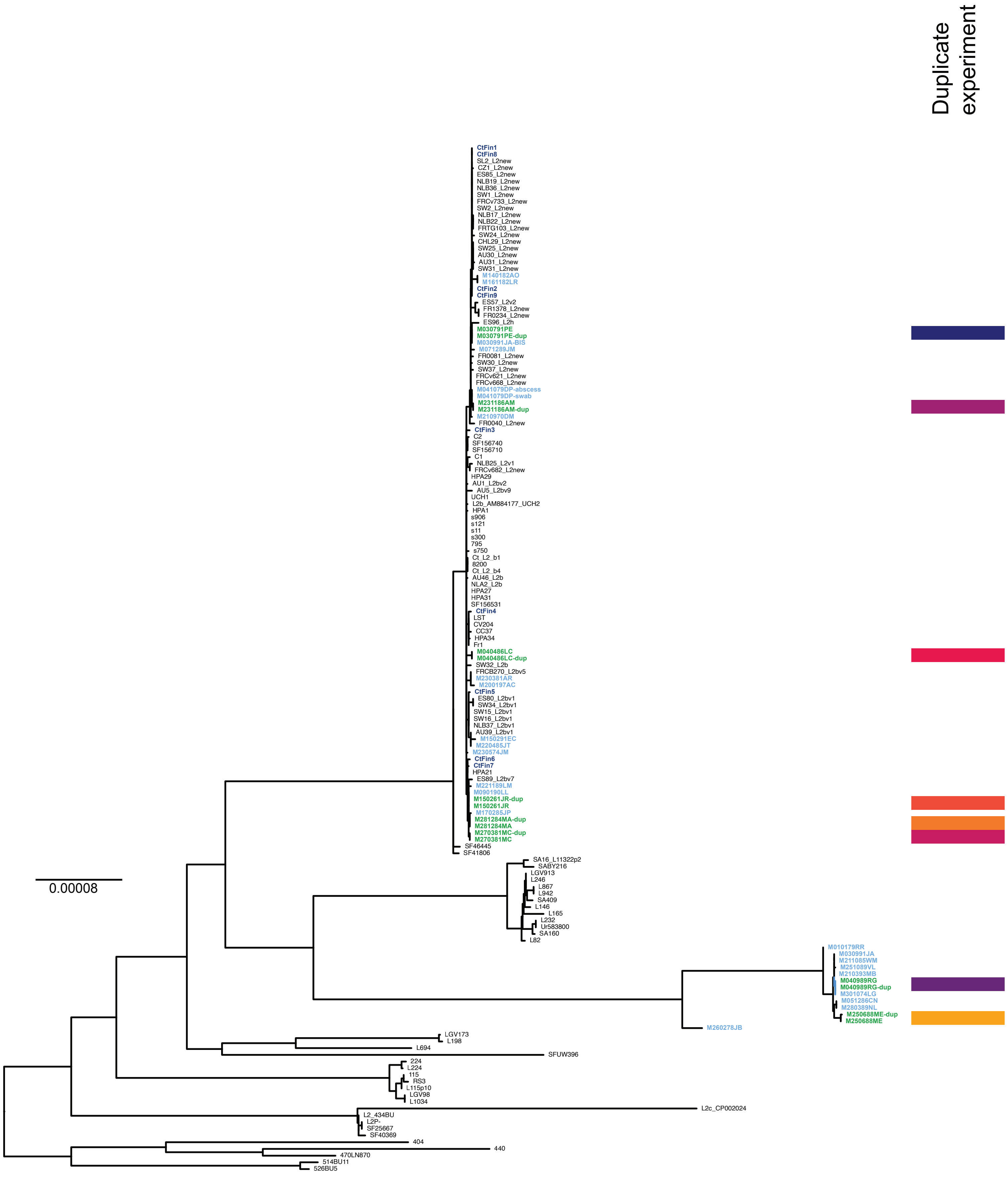

Figure S1. Phylogeny of LGV strains without adjusting for recombination, including duplicate experiments. Duplicate experiments (n=8, to the right of the phylogeny, paired colours) show the robustness of the SureSelect method. Names of genomes from this publication are coloured in the phylogeny in light blue from Argentina and in dark blue from Finland. Names in green are those with identical duplicates ("-dup"). Scale bar refers to a phylogenetic distance of 0.00008 nucleotide substitutions per site.

Figure S2. Recombination-adjusted phylogeny of LGV strains from Argentina, Finland and from previous publications showing identified recombinations. Right of the phylogeny the tracks of identified recombination are shown, with red indicating presence in multiple genomes and blue in single genomes. Above the recombination tracks are the coding sequences of strain L2b/UCH2 (AM884177) and below the tracks, peaks of recombination events. Loci affected (named from AM884177 annotation) are indicated, with those affecting in the Argentinian ompA-genotype L4 clade indicated in pale blue (the latter three only in the 10 strains in the tightly related clade). Newly sequenced isolates are shown in the phylogeny with labels in dark blue (Finland) and pale blue (Argentina). pmp=polymorphic membrane protein, inc=inclusion membrane protein, PZ=plasticity zone.

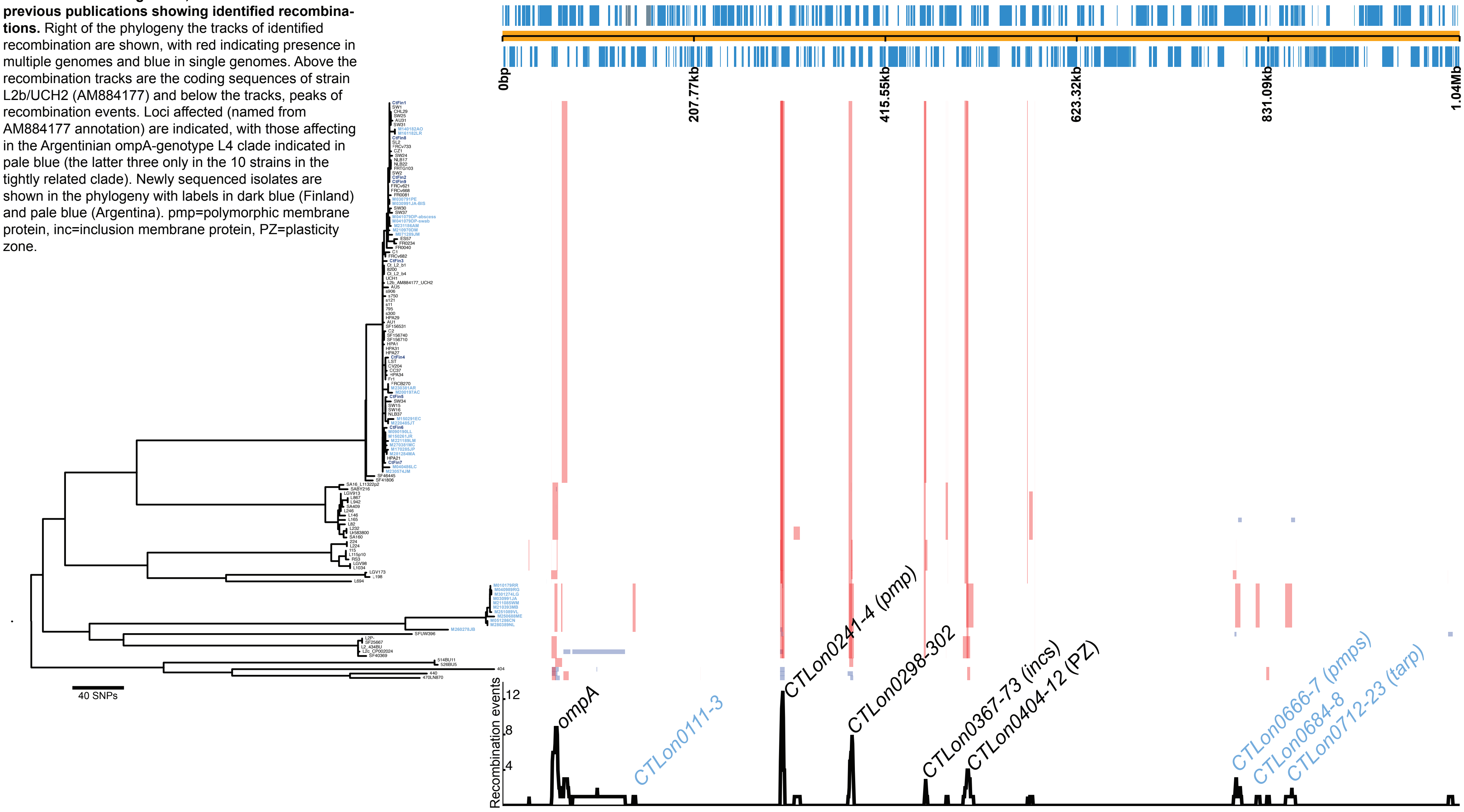

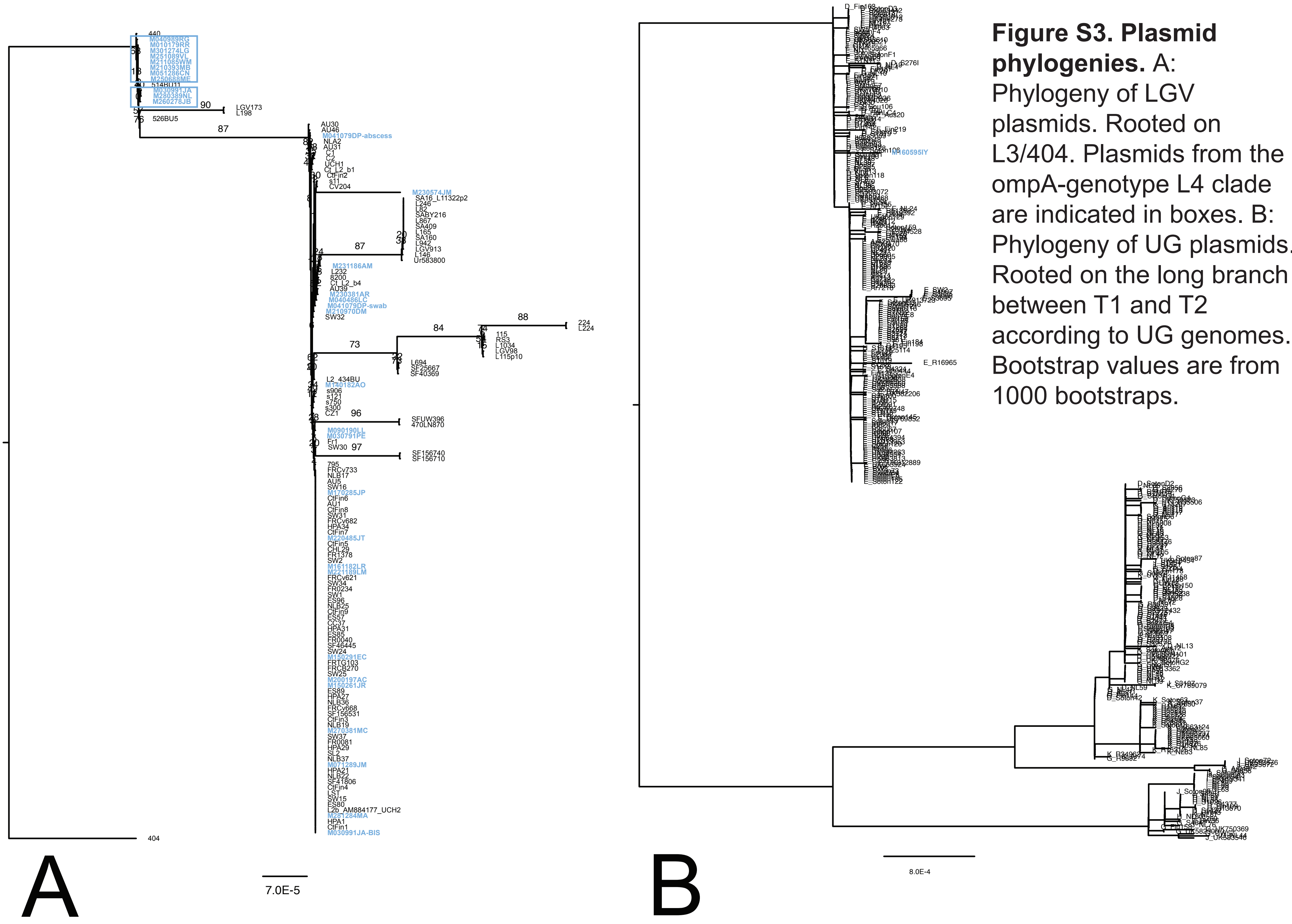

Figure S3. Plasmid phylogenies. A: Phylogeny of LGV plasmids. Rooted on L3/404. Plasmids from the ompA-genotype L4 clade are indicated in boxes. B: Phylogeny of UG plasmids. Rooted on the long branch between T1 and T2 according to UG genomes. Bootstrap values are from 1000 bootstraps.

Table S1. Description of samples included in the study

Sample	Cultured	Sureselect	Duplicate patient sample	Duplicate experiment	Year	Country	Source	Patient gender	MLST	ompA	ompA description (Reference and base changes)	Sample accession	Reads accession	Reads accession -dup sample	Platform
**CtFin1**	Y	N			2011	Finland	rectal swab	M		L2	AM884176	ERS20600757	ERR13385319 (CtFin1-cat)		MiSeq
**CtFin2**	Y	N			2011	Finland	rectal swab	M	58	L2	AM884176	ERS20600758	ERR13385320 (CtFin2-cat)		MiSeq
**CtFin3**	Y	N			2011	Finland	rectal swab	M		L2b	AM884177	ERS20600759	ERR13385321 (CtFin3-cat)		MiSeq
**CtFin4**	Y	N			2011	Finland	rectal swab	M		L2b	AM884177	ERS20600760	ERR13385322 (CtFin4-cat)		MiSeq
**CtFin5**	Y	N			2012	Finland	rectal swab	M	58	L2bv1	JX971936, KC534859	ERS20600761	ERR13385323 (CtFin5-cat)		MiSeq
**CtFin6**	Y	N			2012	Finland	rectal swab	M	58	L2b	AM884177	ERS20600762	ERR13385324 (CtFin6A-cat)		MiSeq
**CtFin7**	Y	N			2012	Finland	rectal swab	M		L2b	AM884177	ERS20600763	ERR13385325 (CtFin7A-cat)		MiSeq
**CtFin8**	Y	N			2013	Finland	rectal swab	M	58	L2	AM884176	ERS20600764	ERR13385326 (CtFin8A-cat)		MiSeq
**CtFin9**	Y	N			2013	Finland	rectal swab	M		L2	AM884176	ERS20600765	ERR13385327 (CtFin9A-cat)		MiSeq
**M010179RR**	N	Y			2020	Argentina	rectal swab	M		L4	HE601950.1 (G268A, T348A, C462T, G471A, A474G, C477T, C594T, A931G, C1017T, A1020C)	ERS20600766	ERR13397787 (M010179RR-ssT4)		NextSeq1000
**M030791PE**	N	Y		1	2018	Argentina	rectal swab	M		L2	AM884176	ERS20600769	ERR13385332 (M030791PE-ssT4)	ERR13397790 (M030791PE-ssT2)	NextSeq1000
**M030991JA**	Y	N	2		2018	Argentina	rectal swab	M		L4v1	HE601950.1 (G268A, T348A, C462T, G471A, A474G, C477T, A485G, C594T, A931G, C1017T, A1020C)	ERS20600770	ERR13385333 (M030991JA-1)		MiSeq
**M030991JA-BIS**	N	Y	2		2018	Argentina	rectal swab	M		L2	AM884176	ERS20600771	ERR13385334 (M030991JA-BIS-ssT2)		NextSeq1000
**M040486LC**	N	Y		5	2019	Argentina	rectal swab	M		L2b	AM884177	ERS20600772	ERR13397791 (M040486LC-ssT2)	ERR13397792 (M040486LC-ssT4)	NextSeq1000
**M040989RG**	N	Y		2	2019	Argentina	rectal swab	M	2	L4	HE601950.1 (G268A, T348A, C462T, G471A, A474G, C477T, C594T, A931G, C1017T, A1020C)	ERS20600774	ERR13397792 (M040989RG-ssT2)	ERR13397795 (M040989RG-ssT2-rep)	NextSeq1000
**M041079DP-abscess**	N	Y	1		2018	Argentina	abscess	M		L2	AM884176	ERS20600775	ERR13397796 (M041079DP-a-ssT2)		NextSeq1000
**M041079DP-swab**	N	Y	1		2018	Argentina	rectal swab	M		L2	AM884176	ERS20600776	ERR13397797 (M041079DP-swab-ssT2)		NextSeq1000
**M051286CN**	N	Y			2021	Argentina	rectal swab	M		L4	HE601950.1 (G268A, T348A, C462T, G471A, A474G, C477T, C594T, A931G, C1017T, A1020C)	ERS20600778	ERR13397802 (M051286CN-ssT4)		NextSeq1000
**M071289JM**	Y	Y			2022	Argentina	rectal swab	M		L2	AM884176	ERS20600779	ERR13397803 (M071289JM-ssT2)		NextSeq1000
**M090190LL**	N	Y			2020	Argentina	rectal swab	M		L2b	AM884177	ERS20600782	ERR13397806 (M090190LL-ssT4)		NextSeq1000
**M140182AO**	N	Y			2023	Argentina	rectal swab	M	58	L2	AM884176	ERS20600784	ERR13397808 (M140182AO-ssT4)		NextSeq1000
**M150261JR**	N	Y		6	2018	Argentina	rectal swab	M		L2bv12	AM884177 (N164D)	ERS20600785	ERR13397809 (M150261JR-ssT2)	ERR13385353 (M150261JR-ssT4)	NextSeq1000
**M150291EC**	Y	N			2019	Argentina	rectal swab	M		L2bv1	JX971936, KC534859	ERS20600786	ERR13385354 (M150291EC)		NextSeq1000
**M161182LR**	N	Y			2023	Argentina	rectal swab	M		L2	AM884176	ERS20600790	ERR13397815 (M161182LR-ssT4)		NextSeq1000
**M170285JP**	Y	N			2019	Argentina	rectal swab	M	58	L2i	AM884177 (G485T)	ERS20600791	ERR13385360 (M170285JP)		NextSeq1000
**M200197AC**	Y	N			2018	Argentina	rectal swab	M		L2bv5	MH253040	ERS20600793	ERR13385362 (M200197AC-4)		MiSeq
**M210393MB**	Y	N			2019	Argentina	rectal swab	M	2	L4	HE601950.1 (G268A, T348A, C462T, G471A, A474G, C477T, C594T, A931G, C1017T, A1020C)	ERS20600794	ERR13385363 (M210393MB-3)		MiSeq
**M210970DM**	N	Y			2021	Argentina	rectal swab	M		L2	AM884176	ERS20600796	ERR13397819 (M210970DM-ssT4)		NextSeq1000
**M211085WM**	Y	N			2018	Argentina	rectal swab	M	2	L4	HE601950.1 (G268A, T348A, C462T, G471A, A474G, C477T, C594T, A931G, C1017T, A1020C)	ERS20600797	ERR13385367 (M211085WM)		NextSeq1000
**M220485JT**	N	Y			2018	Argentina	rectal swab	M	58	L2bv1	JX971936, KC534859	ERS20600798	ERR13397820 (M220485JT-ssT2)		NextSeq1000
**M221189LM**	Y	N			2019	Argentina	rectal swab	M		L2b	AM884177	ERS20601040	ERR13385372 (M221189LM)		NextSeq1000
**M230381AR**	N	Y			2023	Argentina	rectal swab	M		L2bv5	MH253040	ERS20600800	ERR13397824 (M230381AR-ssT2)		NextSeq1000
**M230574JM**	Y	N			2017	Argentina	rectal swab	M		L2b	AM884177	ERS20600801	ERR13385374 (M230574JM)		NextSeq1000
**M231186AM**	N	Y		3	2019	Argentina	rectal swab	M		L2	AM884176	ERS20600802	ERR13397826 (M231186AM-ssT4)	ERR13397825 (M231186AM-ssT2)	NextSeq1000
**M250688ME**	N	Y		8	2018	Argentina	rectal swab	M	2	L4	HE601950.1 (G268A, T348A, C462T, G471A, A474G, C477T, C594T, A931G, C1017T, A1020C)	ERS20600803	ERR13397827 (M250688ME-ssT2)	ERR13397828 (M250688ME-ssT4)	NextSeq1000
**M251089VL**	Y	N			2019	Argentina	rectal swab	M	2	L4	HE601950.1 (G268A, T348A, C462T, G471A, A474G, C477T, C594T, A931G, C1017T, A1020C)	ERS20600804	ERR13385379 (M251089VL-2)		MiSeq
**M260278JB**	N	Y			2021	Argentina	rectal swab	M		L4	HE601950.1 (G268A, T348A, C462T, G471A, A474G, C477T, C594T, A931G, C1017T, A1020C)	ERS20600807	ERR13397832 (M260278JB-pool)		NextSeq1000
**M270381MC**	N	Y		4	2017	Argentina	rectal swab	M	58	L2b	AM884176	ERS20600808	ERR13397835 (M270381MC-ssT2)	ERR13397836 (M270381MC-ssT2-rep)	NextSeq1000
**M280389NL**	N	Y			2022	Argentina	rectal swab	M		L4	HE601950.1 (G268A, T348A, C462T, G471A, A474G, C477T, C594T, A931G, C1017T, A1020C)	ERS20600809	ERR13397837 (M280389NL-ssT2)		NextSeq1000
**M281284MA**	N	Y		7	2018	Argentina	rectal swab	M	58	L2b	AM884176	ERS20600811	ERR13397841 (M281284MA-ssT4)	ERR13397840 (M281284MA-ssT2)	NextSeq1000
**M301274LG**	Y	N			2019	Argentina	rectal swab	M		L4	HE601950.1 (G268A, T348A, C462T, G471A, A474G, C477T, C594T, A931G, C1017T, A1020C)	ERS20600813	ERR13385393 (M301074LG-5)		MiSeq
**M160595IY**	N	Y			2021	Argentina	rectal swab	M		D	ERR027327 (A636T)	ERS20600788	ERR13397812 (M160595IY-ssT4)		NextSeq1000
